# The Influencing Factors of Gender Differences on Mental Burdens in Young Physiotherapists and Occupational Therapist

**DOI:** 10.3390/ijerph18062858

**Published:** 2021-03-11

**Authors:** Su-Jin Lee, Sang In Jung, Myung-Gwan Kim, Eunhee Park, Ae-Ryoung Kim, Chul Hyun Kim, Jong-Moon Hwang, Tae-Du Jung

**Affiliations:** 1 Graduate School of Public Health, Kyungpook National University, Daegu 41944, Korea; sujin89898@gmail.com (S.-J.L.); curein@naver.com (M.-G.K.); 2 Unit of Rehabilitation Therapy, Kyungpook National University Chilgok Hospital, Daegu 41404, Korea; normalman80@gmail.com; 3 Clinical Omics Institute, School of Medicine, Hakjungdong Campus of Kyungpook National University, Daegu 41404, Korea; 4 Department of Rehabilitation Medicine, Kyungpook National University Hospital, Daegu 41944, Korea; ehmdpark@naver.com (E.P.); ryoung20@hanmail.net (A.-R.K.); chgim@knu.ac.kr (C.H.K.); 5 Department of Rehabilitation Medicine, School of Medicine, Kyungpook National University, Jung-gu, Daegu 41944, Korea

**Keywords:** physiotherapist, occupational therapist, burnout syndrome, gender, job stress, job satisfaction

## Abstract

Background: Gender differences in personal discrimination experience, burnout, and job stress among physiotherapists and occupational therapists are considered as associated factors of job loss, poor job quality, high turnover, and economic losses due to the outflow of medical personnel. Thus, the purpose of this study is to analyze the level of burnout, personal discrimination experience, and job stress according to gender differences for young physiotherapists and occupational therapists. Furthermore, we used regression analyses to determine the contribution of gender differences in personal discrimination experience and job stress to self-reported burnout, considering gender and two age groups (younger than 30 years old vs. 30 years old and over). Methods: A total of 325 professional physiotherapists and occupational therapists were part of this study (*n* = 325; male *n* = 131; female *n* = 194. Age: 20–29 years old, *n* = 178; ≥30 years old, *n* = 147). Data were collected using a questionnaire including our three study variables (scales: the Maslach Burnout Inventory (MBI), a modified version of the gender section of the Medical School Graduation Questionnaire from the Association of American Medical Colleges; and the Korean version of the Job Content Questionnaire (JCQ)). Further, data on socio-demographic factors, job-related factors, health-related factors, and subjective job satisfaction were also collected. Results: There were significant positive correlations between job stress and gender differences in personal discrimination experience and job stress and burnout in women 30 years old and over. Personal experience of gender discrimination (β = 0.179, *p* = 0.015) and job stress (β = 0.162, *p* = 0.028) explained a significant 42.4% of the variance in burnout in the case of younger female participants (20–29 years old). However, this model did not explain a significant amount of the variance in burnout in the case of younger male participants (R^2^ = 0.156, *p* = 0.072). Regarding participants aged 30 years and over, our results showed that only job stress (but no personal experience of gender discrimination) was a relevant predictor for both males (β = 0.471, *p* < 0.001) and females (β = 0.373, *p* = 0.001). Conclusion: In this study, female therapists showed higher levels of burnout than male therapists. In particular, personal discrimination experience and job stress significantly contributed to burnout in younger female therapists while job stress was the most relevant predictor variable of burnout for both males (under 30 years old and 30 years old and over) and females in their thirties and beyond. For young female physiotherapists and occupational therapists, safe working environments should be created to reduce work-related mental burdens. It is also necessary to consider policies and regulations that can prevent job stress for therapists and measures that can positively resolve the unavoidable job stress.

## 1. Introduction

The American psychologist Herbert Freudenberger first used the term “burnout” in 1970. He described burnout as becoming exhausted due to experiences of failure, being worn-out, excessive social and physical job demands, or strength or resource demands [1]. Job burnout was eventually defined as a negative psychological reaction caused by an increase in chronic work stress [2]. Three sub-factors describing burnout syndrome were defined by Maslach and Jackson as three major dimensions; namely, emotional exhaustion (EE), depersonalization (DP), cynicism, and lowered perception of personal achievement (PA) [1,3]. First, emotional exhaustion is a major aspect of general exhaustion. Second, depersonalization is the occurrence of negative or cynical attitudes and feelings in the subject. Finally, lowered perception of personal achievement is a negative evaluation of oneself when carrying out tasks.

Physicians, nurses, and health-care professionals have high burnout rates owing to their burden of understanding of diseases and treatment, safety, and high-quality patient care [4,5,6]. Burnout syndromes have been attributed to other causes as follows: Health and social-care professionals have continuous contact and form a high-touch professional relationship with clients in the workplace [2]. This direct and long-term relationship is an important cause leading to severe burnout. Also, health-care professionals who need to make daily contact with patients and who suffer their own physical and mental pain from various conditions of disability have a natural emotional response and set a distance from patients, and this relationship has been reported to be a significant burnout symptom in health professionals in general and in physiotherapists [7,8].

The personality of the physiotherapist or occupational therapist acts as a therapeutic working tool [9,10]. Professionals act as an intervention, providing emotional and physical support to patients as well as dealing with aggressive and depressive patient behavior; this might be overburdening for therapists [7]. In addition, as members of rehabilitation teams for improved health-care services, they expend additional energy in providing services and communicating with co-workers [10].

Many studies abroad have reported higher burnout rates in female medical school students, physicians, nurses, and health-care workers than in males [11,12,13,14,15]. However, while there are differences within individual professions, and among demographic subgroups, the risk factors affecting these women’s burnout index are similar [16]. These risk factors include lack of appropriate role models, difficulty as a dual-career couple, parenting, lack of parity in salary, loss of promotion opportunities, and exposure to higher sexual harassment than men [17,18].

Burnout in health-care professionals may cause increased medical error and decreased job satisfaction [19,20,21]. In 2014, a study examining doctor burnout symptoms in the United States found that 54% of doctors experienced one or more burnout symptoms, 32.8% reported severe fatigue, and 6.5% reported suicide ideation [22]. In particular, the burnout and suicide rates of female doctors were 2.27 times higher than those of the general public in the United States, with women showing higher suicide rates than men [22]. A study found that the relative risk of suicide compared to the general population was 2.5 to 5.7 times higher for female doctors (female doctor relative risk, 2.5–5.1; male doctor relative risk, 1.1–3.4) [23]. In a study examining the relationship between burnout and suicide in Dutch majors, 12% of the 432 majors who experienced burnout reported suicidal thoughts at least once [24]. Suicidal thought was substantially more prevalent in this group (burnout group, 20.5%) compared to the non-burnout group (7.6%) (x^2^ = 182.9, *p* < 0.001).

Burnout is not only associated with suicide, but also causes drug and alcohol abuse, depression [25], and lack of concentration, and this inhibits the quality of medical care and treatment, and given the impact on the formulation of the profession, caused turnover [26,27]. High rates of burnout also hurt employees and the entire organizational group. The absence of personnel and frequent sick leave not only reduce the quality of health care but also burden other team members, which increases the risk of medical error [28,29].

Various studies have been conducted overseas on the burnout status of physiotherapists and occupational therapists [30,31,32]. In one study on the relationship between job stress and quality of life in physiotherapists and occupational therapists, there was no significant correlation between job stress and workload in the quality of life in men [33]. However, in women, there was a positive correlation between them. In domestic studies on burnout of physiotherapists and occupational therapists, gender-related burnouts, job satisfaction, and quality of life have been poorly examined, and gender has only been mentioned as a binary variable [34,35,36].

This study aimed to analyze and compare burnout and risk factors of therapists and occupational therapists through an examination of gender-discriminative personal experiences and job stress that may affect burnout. If there were differences in burnout scores by gender, correlations between the scales would be examined. Moreover, it also examined which demographic factors indicate severe personality discrimination, job stress, and burnout. Therapist’s burnout is an important factor to explore because it is an important factor in lowering job satisfaction and increasing intention to leave. This affects the quality of medical services and patient satisfaction, which leads directly to economic losses due to the outflow of medical personnel [37,38]. Therefore, this study could assist in reducing burnout and job stress among therapists and increasing job satisfaction, by providing evidence that can be used for creating a safe treatment environment, increasing the quality of service, and patient satisfaction.

## 2. Methods

### 2.1. Study Subjects

Physiotherapists and occupational therapists were recruited from various hospitals in several provinces of South Korea between March and May 2019. The researcher directly visited the rehabilitation department of the hospital, where the senior therapist of each hospital’s rehabilitation department agreed to the survey. The researcher explained that participation in this study was to be voluntary, and 325 therapists (131 males and 194 females) agreed to participate in this study.

The researcher explained to the subjects who agreed to participate in the study that this study guaranteed anonymity and subjects were asked to respond first to the socio-demographic and characteristics questions before responding to the burnout, gender-discrimination personal experience, and job stress questionnaire. The first questions contained general information such as gender, age, annual income, and job group as well as items on the type of employment, workplace size, and personal health behaviors such as smoking, drinking, exercising, and leisure time.

The study was cross-sectional and analyzed primary data to understand the relationship between gender-discrimination personal experiences and job stress as factors that affect the burnout degree.

### 2.2. Burnout Symptoms

The Maslach Burnout Inventory (MBI) developed by Maslach and Jackson (1986) was used for this study [3]. The burnout measurement tool consisted of 22 items. At the time of designing the questionnaire, its Cronbach’s alpha value was 0.76. In this study, Cronbach’s alpha value for the burnout measurement tool was 0.719.

This scale consists of three subscales: independent emotional exhaustion, depersonalization, and personal achievement. Emotional exhaustion measures the emotional exhaustion that participants feel while working (nine items), depersonalization measures the level of rapport with clients (five items), and personal achievement measures the achievement of workers (eight items).

A 5-point Likert scale ranging from ‘disagree’ (1) to ‘agree’ (5) was used to score each item, and the scores measured for each sub-concept were summed and divided by the number of questions, respectively. The mean score was used in this study.

Personal achievement, which is a sub-variable of burnout, was reverse-coded in this study, i.e., lower scores on PA indicated higher levels of burnout. These higher scores on emotional exhaustion and depersonalization indicated a more severe burnout state.

### 2.3. Gender-Specific Personal Experience

We modified the gender section of the Medical School Graduation Questionnaire from the Association of American Medical Colleges (AAMC) to match our domestic Korean situation to investigate personal experiences according to gender [39].

The revised questionnaire used in this study included 12 items on the perception of and attitude towards personal experiences of gender discrimination. These items were rated on a 4-point scale from not at all (0) to often (3). Scores on this scale range from 0 to 36, where higher scores indicate more experiences of individual gender-based discrimination. For this study, the Cronbach’s alpha for the present tool was 0.868.

### 2.4. Job Stress

To evaluate the risk of developing work-related stress, the 22-item Korean version JCQ (Job Content Questionnaire), which consists of three scales, was administered [40,41].

The 22-item Korean version of JCQ is a questionnaire adapted to the working environment in Korea and its validity has been verified [42]. In many studies abroad, JCQ-22 is actively used according to each country’s situation and work environment [43,44].

The three scales are “decision latitude” including skill discretion (six items) and decision authority (three items), “psychological job demands” (five items), and “social support” including coworker support (four items) and supervisor support (four items). The higher scores indicated a stressful experience at work. Items in the scales are scored using a Likert scale in which 0 indicates that the respondent strongly agrees, and 3 indicates that the respondent strongly disagrees. In this study, Cronbach’s alpha value of the JCQ measurement tool was 0.785.

### 2.5. Statistical Analysis

All statistical analyses were carried out using the open-source statistical software R 3.6.0. To analyze the general characteristics, working characteristics, and health behaviors of the subjects, a frequency analysis was conducted. Independent *t*-test and ANOVA were performed to identify gender-specific personal experiences, job stress, and burnout. Pearson correlation coefficients were analyzed by gender and age for gender-specific personal experiences, job stress, and burnout. To identify the factors affecting burnout, multiple regression analysis that analyzed gender-specific personal experience and job stress, and multiple regression analysis with general characteristics, working characteristics, and health behavior was performed, after dividing the study sample according to gender and age.

### 2.6. Ethical Considerations

The protocol used in this study was approved by the institutional review board of the hospital (IRB No. 2019-03-006), and written informed consent was formally obtained from all the participants.

## 3. Results

### 3.1. Sociodemographic Characteristics of Participants

The sociodemographic characteristics of the participants in this study are shown in Table 1 and Table 2. Of the 325 participants in this study, 178 (54.8%) were 20–29 years old and 147 (45.2%) were over 30 years old. The distribution according to professional groups was 216 physiotherapists (66.5%) and 109 occupational therapists (33.5%).

#### 3.1.1. Differences between Men and Women in Personal Experiences of Gender Discrimination, Job Stress, and Burnout

Gender was found to be significantly associated with personal experiences of gender discrimination (*t* = −9.32, *p* < 0.001), job stress (*t* = 3.08, *p* < 0.002), and burnout (*t* = −5.15, *p* < 0.001) indicating that females experienced significantly higher levels of gender discrimination and burnout compared to their male counterparts. Males experienced significantly higher levels of job stress than females (*t* = 3.08, *p* = 0.002). Concerning age, the burnout rate of those between 20 and 29 years old was higher than that for over-30-year-olds (*t* = 5.06, *p* < 0.001) (Table 1).

#### 3.1.2. Burnout Indices by Sociodemographic Characteristics of Rehabilitation Professionals

The degree of burnout was divided into three subscales. First, female therapists had significantly higher degrees of emotional exhaustion than male therapists (*t* = −4.21, *p* < 0.001), and women between 20 and 29 years old showed higher levels of emotional exhaustion than those who were over 30 years old (*t* = 4.31, *p* < 0.001). The group with an annual income of KRW 20 million to KRW 30 million had a higher score than those with an annual income of KRW 30 million or more (F = 11.15, *p* < 0.001), the group working at rehabilitation hospitals showed the highest EE score (F = 8.32, *p* < 0.001). Lack of sleep time had a higher score than enough time (*t* = 5.83, *p* < 0.001), and no leisure time had a higher score than having leisure time group (F = 3.77, *p* = 0.024). Second, females showed significantly higher degrees of depersonalization than males (*t* = −2.63, *p* = 0.009), and women between 20 and 29 years old showed higher levels of depersonalization than those who were over 30 years old (*t* = 3.32, *p* = 0.001). The group with an annual income of less than KRW 30 million had a higher score for DP than high-income groups (F = 10.32, *p* < 0.001), the group working at a rehabilitation hospital or smaller-size hospital had a higher score than those working in a general hospital (F = 16.18, *p* < 0.001), and the group with no time to have leisure time had a higher score than the other groups (F = 4.45, *p* = 0.012).

Third, females have significantly higher degrees of personal achievement than males (*t* = −4.37, *p* < 0.001), and women between 20 and 29 years old showed higher levels of personal achievement than those who were over 30 years old (*t* = 3.90, *p* < 0.001). The group with an annual income of less than KRW 30 million had a higher PA score than the others (F = 15.32, *p* < 0.001), the group working at a rehabilitation hospital and convalescent hospital had a higher score than those working at a general hospital (F = 19.66, *p* < 0.001), and the group with no leisure time had a higher score than the others (F = 3.46, *p* = 0.033).

There was no difference in burnout indices between physiotherapists and occupational therapists (Table 2).

#### 3.1.3. Correlation of the Gender-Discrimination Personal Experience, Job Stress, and Burnout

Pearson correlation analysis was performed to investigate the relationship between gender-discrimination personal experience, job stress, and burnout according to gender and age. Results showed a significant correlation between burnout and job stress in females of all ages. However, especially in females over 30 years-of-age, there was a higher positive correlation (*r* = 0.56, *p* < 0.001). In the case of 20–29-year-old women, gender-discrimination personal experience, and burnout showed a significant but weak positive correlation (*r* = 0.28, *p* = 0.001) (Table 3). In the case of males, there was a significant positive correlation between burnout and job stress among those above 30 years-of-age (*r* = 0.44, *p* < 0.001) (Table 3).

#### 3.1.4. Multiple Regression Analysis According to Burnout, Gender-Specific Personal Experience, and Job Stress

To identify the factors influencing burnout, multiple regression analysis was performed on burnout and gender-discrimination personal experience, and burnout and job stress, based on age and gender. This model showed that personal experience of gender discrimination (β = 0.179, *p* = 0.015) and job stress (β = 0.162, *p* < 0.028) explained a significant 42.4% of the variance of burnout in the case of younger female participants (20–29 years old). However, the result of this model did not show a significant amount of the variance in burnout in the case of younger male participants (R^2^ = 0.156, *p* = 0.072). The results showed that solely job stress was a relevant predictor for both males (β = 0.471, *p* < 0.001) and females (β = 0.373, *p* = 0.001). Regarding control variables, gender differences were also found for physiotherapists and occupational therapists. Job self-respect (β = −0.469, *p* < 0.001) and education level (β = −0.169, *p* = 0.028) negatively contributed to burnout reported by younger females. Job self-respect (β = −0.229, *p* = 0.018) also decreased burnout in the group of males in their 30’s and over (Table 4).

## 4. Discussion

This was a cross-sectional study that measured primary data to quantitatively evaluation the relationship between gender-discrimination personal experiences and job stress as factors that affect the burnout degree. This study of physiotherapists and occupational therapists showed that women scored higher on gender-based personal experience of discrimination (M = 0.51, *t* = −9.32, *p* < 0.001) and burnout (M = 2.42, *t* = 5.15, *p* < 0.001) than men. Another study found that female physicians (79%) had higher burnout rates than male physicians (62%) [45]. It has been reported that gender has a significant association with burnout; in one study, female oncologists reported high levels of burnout than males (female, 2.59 ± 1.69; male, 1.84 ± 1.6; *t* = −3.05; *p* < 0.01) [46]. A study of burnout levels using the Burnout Inventory Scale by Steuden and Okla for physiotherapists in Poland, showed significantly high burnout in female therapists, related to their low life satisfaction [47]. There are various reasons for gender differences in burnout rates. Women have to cater to the emotional needs of their families and, simultaneously, deal with multiple other tasks and challenges arising from their roles in families [45]. Other reasons include exclusion from promotion to leadership positions [48] and lower salaries compared to their male counterparts [48,49]. However, studies of US neurologists showed that gender is not an independent predictor of burnout for high burnout rates and low job satisfaction among female neurologists [50]. Additionally, burnout is experienced differently in men and women; women experience higher emotional exhaustion while men experience a higher risk of depersonalization [51]. Another study showed that burnout in women and men may appear differently [7]. In women, the higher risk and the tendency to get involved in the patient’s problems leads to a higher risk factor for emotional exhaustion. However, several authors note that the risk of developing burnout is not related to gender [52,53].

In our study, women had higher burnout levels in emotional exhaustion (*t* = −4.21, *p* < 0.001), depersonalization (*t* = −2.63, *p* = 0.009), and personal achievement (*t* = −4.37, *p* < 0.001) on the burnout scale than men. In a study of burnout symptoms among therapists, women were scored significantly higher in personal achievement than men (F = 3.98, *p* = 0.048), and depersonalization was higher in men than in women [54]. However, there was no gender difference in emotional exhaustion (F = 2.58, *p* = 0.111). Another study found that male therapists showed significantly higher burnout (*p* < 0.049) in depersonalization than women, but gender was not a determinant of burnout syndrome [55]. It was found that men experience higher depersonalization than women do [56]. The differences in burnout are due to gender differences in social expectations, and therapist’s attitudes towards patients such as an approach without humanity and dehumanization of patients due to traits such as strength, independence, occupational achievement, and invulnerability of men [57].

In our study, female therapists in their twenties showed higher burnout scores on all subscales of burnout than those who over 30 years-of-age (emotional exhaustion, *t* = 4.31, *p* < 0.001; depersonalization, *t* = 3.32, *p* = 0.001; personal achievement, *t* = 3.90, *p* < 0.001). These findings coincide with the results of previous studies that found that younger and less experienced therapists have a higher risk of job-related burnout [2,58], and experienced workers cope with stress better and are less likely to be at risk of burnout. Another study of burnout in physiotherapists and occupational therapists found that there was a weak but significant correlation between emotional exhaustion and age (*r* = −1.90, *p* < 0.01) [59]. However, according to a study, there was no age-difference in the degree of burnout [54]. Physiotherapists who have less than five years of work experience might be at greater risk of burnout due to difficulties in meeting patient needs and making work-related decisions [59].

In our study, the positive correlation between job stress and burnout in women aged 20–29 years (*r* = 0.34, *p* < 0.001), and both job stress and burnout in both women and men over 30 years-of-age (male: *r* = 0.43, *p* < 0.001; female: *r* = 0.56, *p* < 0.001). This result is similar to the results of Canara’s study that women among healthcare workers show a higher degree of burnout than men [60].

Burnout is likely to develop due to the accumulation of chronic job stress, which makes active problem solving difficult [61]. Burnout symptoms caused by job stresses often lead to apathy, cynicism, and rigidity, as well as a lack of empathy and compassion for co-workers and their clients. Such dissatisfaction due to job stress causes discoloration of purpose and meaning to their profession [61].

In a UK study, work-related stress was related to the most widely-known factor, excessive workload, along with factors such as harassment, shift work, sexual harassment or racism, staff reductions, and departmental shifts [62]. In our study, we found a significant correlation between gender discrimination and burnout symptoms in women aged 20–29 (*r* = 0.277, *p* = 0.001). This study was conducted with a modified questionnaire, which was based on the “personal experiences with negative behaviors” section [39] of the questionnaire for medical graduates of the Association of American Medical Colleges (AAMC) to investigate gender-based personal experiences of female therapists at the workplace. This showed that female participants have “had more than one experience of being discriminated against by patients because of their gender” (Q9: 65% of all respondents). More than once, they were asked to change therapists by their patients, and their caregivers (Q10: 47% of the total respondents) showed a higher response rate than the males (Q9: *t* = −10.88, *p* < 0.001; Q10: *t* = −6.35, *p* < 0.001). As a result, female participants showed higher response rates in ‘I have had more than one experience of discrimination against patients because of my gender’ (Q9: 65% of all female respondents), ‘I have been asked to change the therapist by patients and their care-giver because of my gender (Q10: 47% of all female respondents)’ than males (Q9: *t* = −10.88, *p* < 0.001; Q10: *t* = −6.35, *p* < 0.001). The study by Aisha Yaghmour et al. showed the challenge experienced by female physicians [63]. In that study, female physicians with single marital status and lower level of training duration responded that they experienced gender discrimination, dissatisfaction, and drop-out rates in their workplace [63].

The limitations of this study are as follows. First, the survey method used self-report questionnaires, wherein responses may be affected by recall bias. Second, items reconstructed according to the domestic situation were used, such as Maslach and Jackson and Association of American Medical Colleges (AAMC) [3,39]. Therefore, this questionnaire is limited in direct comparison with previous studies comparing the degree of burnout and sexual harassment in health-care professions. Third, our participants were not randomly selected but were a sample of convenience. A study found that voluntary participants generally had higher motivation than non-volunteers, and there is less variability with such participants’ data [64].

Differences between the results of our study and those of previous studies were found in burnout, job stress, and gender-discrimination personal experience according to a specific gender and specific age group among physiotherapists and occupational therapists. We were able to identify several factors that lower their work efficiency and achievement concerning jobs through quantitative evaluation of the level of burnout, gender-discrimination personal experience, and job stress, which were found more significantly by specific sex and age in physiotherapists and occupational therapists.

## 5. Conclusions

In this study, the degree of burnout in therapists and occupational therapists was examined, and there were significant differences in burnout degree according to gender, and the correlation between burnout subscales according to age and gender was compared. Based on the results of this study, follow-up studies are needed to investigate the relationship between various demographic factors and employment status factors of therapists in rehabilitation medicine and a comparison between health-care worker groups by gender. Regardless of gender, setting meaningful treatment goals for patients and achieving job satisfaction to implement the interventions, and having emotional investment to reduce work-related stress and burnout should be essential for improving teamwork and quality of care in the work-place [65].

## Figures and Tables

**Table 1:** General characteristics of rehabilitation professionals in gender-discrimination personal experience, job stress and burnout scale (MBI).

Variable	Frequency	%	Gender-Discrimination Personal Experience		Job Stress		MBI	
			M (SD)	*t* or F (*p*) Scheffe	M (SD)	*t* or F (*p*) Scheffe	M (SD)	*t* or F (*p*) Scheffe
Sex								
Male	131	40.3	0.13 (0.25)	−9.316 **	1.88 (0.28)	3.077 **	2.15 (0.48)	−5.152 **
Female	194	59.7	0.51 (0.49)	(<0.001)	1.78 (0.24)	(0.002)	2.42 (0.45)	(<0.001)
Age								
20–29	178	54.8	0.39 (0.46)	0.946	1.79 (0.24)	−1.921	2.45 (0.41)	5.061 **
≥30	147	45.2	0.34 (0.44)	(0.345)	1.85 (0.28)	(0.056)	2.18 (0.49)	(<0.001)
Annual income (KRW)								
≥50 million ^a^	26	8.0	0.13 (0.26)	5.691 **	1.93 (0.31)	3.424 *^‡^	1.91 (0.54)	18.599 **
<50–≥30 million ^b^	86	26.5	0.30 (0.40)	(0.001)	1.86 (0.29)	(0.018)	2.11 (0.47)	(<0.001)
<30–≥20 million ^c^	184	56.6	0.44 (0.48)	a < c	1.79 (0.24)	-	2.45 (0.43)	a,b < c,d
<20 million ^d^	29	8.9	0.23 (0.40)		1.83 (0.24)		2.38 (0.39)	
Hospital size								
General hospital ^a^	98	30.2	0.24 (0.38)	5.315 **	1.88 (0.27)	6.069 **	2.05 (0.49)	22.683 **
Rehabilitation hospital ^b^	212	65.2	0.40 (0.46)	(0.005)	1.81 (0.26)	(0.003)	2.42 (0.43)	(<0.001)
Convalescent hospital etc. ^c^	15	4.6	0.51 (0.51)	a < c	1.65 (0.19)	a,b > c	2.48 (0.39)	a < b,c
Job group								
Physiotherapist	216	66.5	0.35 (0.44)	−0.385	1.81 (0.25)	−1.557	2.32 (0.49)	0.641
Occupational therapist	109	33.5	0.37 (0.46)	(0.701)	1.85 (0.29)	(0.120)	2.29 (0.46)	(0.522)
Employment type								
Regular worker	257	79.1	0.34 (0.43)	−1.568	1.83 (0.27)	1.112	2.30 (0.48)	−0.682
Irregular worker	68	20.9	0.44 (0.52)	(0.120)	1.79 (0.25)	(0.267)	2.34 (0.49)	(0.496)
Smoking								
Smoker	42	12.9	0.17 (0.31)	−3.971 **	1.89 (0.28)	1.855	2.15 (0.53)	−2.307 *
Non-smoker	283	87.1	0.39 (0.46)	(<0.001)	1.81 (0.26)	(0.065)	2.33 (0.47)	(0.022)
Drinking								
Alcohol drinker	234	72.0	0.38 (0.47)	1.231	1.83 (0.26)	0.405	2.28 (0.47)	−1.563
Non-drinker	91	26.0	0.31 (0.40)	(0.219)	1.81 (0.27)	(0.686)	2.38 (0.49)	(0.119)
Exercise								
≥5 days a week	22	6.8	0.27 (0.39)	0.453	1.88 (0.24)	0.639	2.26 (0.51)	0.695
1–4 days a week	196	60.3	0.37 (0.44)	(0.636)	1.82 (0.26)	(0.529)	2.29 (0.50)	(0.500)
Does not exercise	107	32.9	0.36 (0.47)		1.81 (0.27)		2.35 (0.43)	
Sleep								
Enough	174	53.5	0.29 (0.42)	−2.750 **	1.83 (0.27)	0.319	2.21 (0.50)	−4.174 **
Lack	151	46.5	0.43 (0.47)	(0.006)	1.82 (0.25)	(0.750)	2.42 (0.43)	(<0.001)
Has leisure time								
Yes	240	73.8	0.37 (0.45)	0.276	1.84 (0.27)	4.613 *^‡^	2.26 (0.47)	5.495 **
No	60	18.5	0.33 (0.47)	(0.759)	1.73 (0.23)	(0.011)	2.49 (0.43)	(0.005)
Does not rest at weekend	22	7.7	0.32 (0.41)		1.85 (0.27)		2.33 (0.55)	
Total	325	100.0	0.36 (0.45)		1.82 (0.26)		2.31 (0.48)	

MBI, Maslach Burnout Inventory; Independent *t*-test and ANOVA, * *p* < 0.05, ** *p* < 0.01; ^‡^ Pairs of variables containing significant differences in the Tukey test.

**Table 2:** Burnout indices by sociodemographic characteristics of rehabilitation professionals.

Variable	Frequency	%	EE		DP		PA	
			M (SD)	*t* or F (*p*) Scheffe	M (SD)	*t* or F (*p*) Scheffe	M (SD)	*t* or F (*p*) Scheffe
Sex								
Male	131	40.3	1.78 (0.71)	−4.205 **	1.15 (0.62)	−2.634 **	3.19 (0.56)	−4.374 **
Female	194	59.7	2.10 (0.62)	(<0.001)	1.33 (0.60)	(0.009)	3.46 (0.53)	(<0.001)
Age								
20–29	178	54.8	2.12 (0.62)	4.311 **	1.38 (0.58)	3.317 **	3.50 (0.49)	3.898 **
≥30	147	45.2	1.79 (0.69)	(<0.001)	1.15 (0.58)	(0.001)	3.25 (0.56)	(<0.001)
Annual income (KRW)								
≥50 million ^a^	26	8.0	1.75 (0.68)	11.148 **.	0.82 (0.76)	10.324 **.	2.78 (0.54)	15.315 **
<50–≥30 million ^b^	86	26.5	1.67 (0.70)	(<0.001)	1.09 (0.58)	(<0.001)	3.24 (0.58)	(<0.001)
<30–≥20 million ^c^	184	56.6	2.13 (0.61)	a,b < c	1.39 (0.56)	a < c,d	3.45 (0.51)	a < b,c,d
<20 million ^d^	29	8.9	1.98 (0.58)		1.25 (0.60)		3.54 (0.41)	
Hospital Size								
General hospital ^a^	98	30.2	1.74 (0.67)	8.302 **	0.98 (0.58)	16.186 **	3.08 (0.57)	19.664 **
Rehabilitation hospital ^b^	212	65.2	2.07 (0.66)	(<0.001)	1.37 (0.59)	(<0.001)	3.46 (0.51)	(<0.001)
Convalescent hospital etc. ^c^	15	4.6	2.02 (0.54)		1.49 (0.59)	a < b,c	3.63 (0.50)	a < b,c
Job group								
Physiotherapist	216	66.5	2.00 (0.69)	1.237	1.30 (0.64)	1.869	3.32 (0.58)	−1.441
Occupational therapist	109	33.5	1.90 (0.63)	(0.217)	1.17 (0.55)	(0.063)	3.41 (0.50)	(0.151)
Employment type								
Regular worker	257	79.1	1.97 (0.67)	−0.046	1.23 (0.60)	−1.651	3.34 (0.56)	−0.421
Irregular worker	68	20.9	1.97 (0.68)	(0.963)	1.36 (0.64)	(0.100)	3.38 (0.55)	(0.674)
Smoking								
Smoker	42	12.9	1.78 (0.79)	−1.689	1.16 (0.64)	−1.122	3.19 (0.61)	−2.022
Non-smoker	283	87.1	2.00 (0.65)	(0.097)	1.27 (0.61)	(0.263)	3.38 (0.54)	(0.044)
Drinking								
Alcohol drinker	234	72.0	1.94 (0.67)	−1.342	1.23 (0.59)	−1.070	3.33 (0.54)	−1.142
Non-drinker	91	26.0	2.05 (0.68)	(0.180)	1.31 (0.66)	(0.285)	3.41 (0.59)	(0.254)
Exercise								
≥5 days a week	22	6.8	1.81 (0.75)	0.971	1.32 (0.66)	0.547	3.34 (0.59)	2.040
1–4 days a week	196	60.3	1.96 (0.68)	(0.380)	1.28 (0.62)	(0.579)	3.30 (0.59)	(0.132)
Does not exercise	107	32.9	2.02 (0.63)		1.21 (0.59)		3.44 (0.48)	
Sleep								
Enough	174	53.5	1.78 (0.70)	5.825 **	1.20 (0.64)	1.665	3.38 (0.55)	0.984
Lack	151	46.5	2.19 (0.57)	(<0.001)	1.32 (0.58)	(0.097)	3.32 (0.56)	(0.326)
Has leisure time								
Yes	240	73.8	1.91 (0.68)	3.770 *^‡^	1.20 (0.61)	4.449 *^‡^	3.32 (0.55)	3.459 *^‡^
No	60	18.5	2.15 (0.56)	(0.024)	1.46 (0.60)	(0.012)	3.51 (0.51)	(0.033)
Does not rest at weekend	22	7.7	2.12 (0.73)		1.30 (0.57)		3.23 (0.64)	
Total	325	100.0	1.97 (0.67)		1.26 (0.61)		3.35 (0.56)	

EE, Emotional exhaustion; DP, depersonalization; PA, personal achievement; independent *t*-test and ANOVA, * *p* < 0.05, ** *p* < 0.01; ^‡^ Pairs of variables containing significant differences in the Tukey test.

**Table 3:** Correlation of the gender-discrimination personal experience, job stress, and MBI.

Variable		Male		Female	
		Gender-Discrimination Personal Experience	Job Stress	Gender-Discrimination Personal Experience	Job Stress
20–29	Job stress	−0.057 (0.700)	1	0.092 (0.267)	1
	MBI	0.140 (0.344)	0.187 (0.202)	0.277 (0.001) **	0.343 (<0.001) **
≥30	Job stress	0.029 (0.812)	1	0.192 (0.174)	1
	MBI	0.232 (0.055)	0.443 (<0.001) **	0.216 (0.125)	0.559 (<0.001) **

MBI, Maslach Burnout Inventory; Pearson correlation coefficients were analyzed by gender and age for gender specificity; ** *p* < 0.01.

**Table 4:** Regression analyses explaining burnout.

Variable		Control Estimation Male			Control Estimation Female		
		B	β	*p*	B	β	*p*
20–29	(constant)	3.604		<0.001	3.528		<0.001
	Year income	−0.002	−0.003	0.986	−0.105	−0.111	0.129
	Education level	−0.001	−0.002	0.991	−0.098	−0.169	0.028
	Marital status—married (ref: single)	−0.056	−0.025	0.881	−0.049	−0.030	0.686
	Hospital size	−0.175	−0.176	0.237	−0.008	−0.008	0.915
	Job self-respect	−0.136	−0.174	0.280	−0.309	−0.469	<0.001
	Sleep	0.039	0.041	0.794	0.028	0.037	0.612
	Has leisure time	−0.138	−0.168	0.273	−0.081	−0.131	0.079
	Gender discrimination Personal experience	0.551	0.282	0.056	0.138	0.179	0.015
	Job stress	0.609	0.360	0.019	0.275	0.162	0.028
Model fit		F = 1.963, *p* = 0.072 R^2^ = 0.317, Adj R^2^ = 0.156			F = 9.816, *p* < 0.001 R^2^ = 0.424, Adj R^2^ = 0.381		
≥30	(constant)	3.597		<0.001	4.216		<0.001
	Year income	−0.068	−0.106	0.280	−0.134	−0.189	0.134
	Education level	−0.023	−0.034	0.708	−0.073	−0.112	0.321
	Marital status—married (ref: single)	−0.107	−0.114	0.244	−0.151	−0.140	0.188
	Hospital size	−0.126	−0.133	0.177	−0.190	−0.175	0.166
	Job self-respect	−0.182	−0.229	0.018	−0.052	−0.054	0.632
	Sleep	0.135	0.144	0.125	0.200	0.184	0.083
	Has leisure time	−0.126	−0.171	0.085	−0.004	−0.005	0.960
	Gender discrimination Personal experience	0.075	0.040	0.661	0.083	0.076	0.470
	Job stress	0.758	0.471	<0.001	0.715	0.373	0.001
Model fit		F = 6.709, *p* ≤ 0.001 R^2^ = 0.456, Adj R^2^ = 0.388			F = 8.450, *p* ≤ 0.001 R^2^ = 0.594, Adj R^2^ = 0.524		

## Data Availability

The data presented in this study are available on reasonable request from the corresponding author. The data are not publicly available due to ethical requirements.
